# Rapid grain boundary diffusion in foraminifera tests biases paleotemperature records

**DOI:** 10.1038/s43247-023-00798-2

**Published:** 2023-04-27

**Authors:** Arthur Adams, Damien Daval, Lukas P. Baumgartner, Sylvain Bernard, Torsten Vennemann, Deyanira Cisneros-Lazaro, Jarosław Stolarski, Alain Baronnet, Olivier Grauby, Jinming Guo, Anders Meibom

**Affiliations:** 1 Laboratory for Biological Geochemistry, School of Architecture, Civil and Environmental Engineering, École Polytechnique Fédérale de Lausanne (EPFL), CH-1015 Lausanne, Switzerland; 2 ISTerre, Université Grenoble Alpes, Université Savoie Mont Blanc, CNRS, IRD, IFSTTAR, 38058 Grenoble, France; 3 Institute of Earth Surface Dynamics, University of Lausanne, CH-1015 Lausanne, Switzerland; 4 Museum National d’Histoire Naturelle, Sorbonne Université, CNRS UMR 7590, IMPMC, 75005 Paris, France; 5 Institute of Paleobiology, Polish Academy of Sciences, PL-00-818 Warsaw, Poland; 6 CNRS, CINaM, Aix-Marseille Université, 13009 Marseille, France; 7 Center for Advanced Surface Analysis, Institute of Earth Science, University of Lausanne, CH−1015 Lausanne, Switzerland

**Keywords:** Palaeoclimate, Geochemistry, Palaeoceanography, Biogeochemistry

## Abstract

The oxygen isotopic compositions of fossil foraminifera tests constitute a continuous proxy record of deep-ocean and sea-surface temperatures spanning the last 120 million years. Here, by incubating foraminifera tests in ^18^O-enriched artificial seawater analogues, we demonstrate that the oxygen isotopic composition of optically translucent, i.e., glassy, fossil foraminifera calcite tests can be measurably altered at low temperatures through rapid oxygen grain-boundary diffusion without any visible ultrastructural changes. Oxygen grain boundary diffusion occurs sufficiently fast in foraminifera tests that, under normal upper oceanic sediment conditions, their grain boundaries will be in oxygen isotopic equilibrium with the surrounding pore fluids on a time scale of <100 years, resulting in a notable but correctable bias of the paleotemperature record. When applied to paleotemperatures from 38,400 foraminifera tests used in paleoclimate reconstructions, grain boundary diffusion can be shown to bias prior paleotemperature estimates by as much as +0.86 to −0.46 °C. The process is general and grain boundary diffusion corrections can be applied to other polycrystalline biocarbonates composed of small nanocrystallites (<100 nm), such as those produced by corals, brachiopods, belemnites, and molluscs, the fossils of which are all highly susceptible to the effects of grain boundary diffusion.

## Abstract (web summary)

The paleoseawater temperature record from the oxygen isotope compositions of fossil foraminifera tests may be biased by up to about 1 °C due to grain-boundary diffusion alone, according to isotope exchange experiments on foraminifera tests.

## Introduction

Oxygen isotope ratios of calcitic foraminifera tests in ocean sediments constitute the most detailed continuous proxy record of deep-water and sea-surface temperatures over the last 120 million years (Ma)^1^. Apart from the effects of biogenic fractionation (i.e., “vital effects”), these oxygen isotope compositions should reflect only the temperature and oxygen isotopic composition of the ambient seawater in which they lived^2^. In the fossil record, this relationship holds as long as the individual tests selected for analysis have not been isotopically altered during their burial in the sediment column through processes collectively known as diagenesis. Despite early concerns^3,4^, the potential impact of diagenesis on foraminifera was not fully appreciated in early work^5–7^, but today many anomalous paleoseawater temperatures have been attributed to recrystallized foraminifera tests, and consequently those early data have been excluded from more recent paleoseawater temperature records^8–11^. To limit diagenetic bias in the paleotemperature record, foraminifera tests are commonly screened by optical microscopy (and sometimes by scanning electron microscopy), to determine if they are “glassy”, i.e., visually pristine, or “frosty” and hence assumed to be diagenetically altered^8,12^. Only “glassy” foraminifera tests are used to reconstruct ocean paleoseawater temperatures, thereby minimizing diagenetic bias from impacting the paleotemperature record.

Recently, this premise has been challenged. Minerals considered stable under ambient conditions can be isotopically and chemically altered through interactions with aqueous fluids, even at low temperatures and without any visible modification to their ultrastructures. This process is coined “stable mineral recrystallization” and has been generally ascribed to dissolution-precipitation reactions^13–16^. In addition, diffusive processes are capable of modifying the bulk isotopic compositions of biocarbonates without any resulting ultrastructural changes, thereby measurably biasing paleotemperature estimations^17^. Until now, the effect of such low-temperature isotope diffusion has generally been ignored, perhaps because of the well-established paradigm that such slow processes cannot substantially alter the isotopic compositions of large (>1 µm) crystals with relatively low surface-to-volume ratios^2,3,18^.

However, biogenic carbonates differ from abiotic minerals with regard to ultrastructure, trace element compositions, and fine-scale, composite organic-mineral interactions. Biogenic carbonates are often perforated by micrometer-sized pores, cut by organic lineaments, and—at the ultrastructural level—composed of 50–200 nm subspherical nanocrystallites interwoven with an inter- and intracrystalline matrix rich in organic molecules^19–24^. Calcitic foraminiferal tests are good examples of such a heterogeneous structural organization. In addition, they are compositionally heterogeneous^25,26^ with concentration bands rich in magnesium, strontium, sodium, and other trace elements creating regions of relatively higher calcite solubility^27,28^ that are hence also more susceptible to recrystallization. This complexity is further complicated by phylogeny, which separates foraminifera into several groups with distinct ultrastructures, crystalline morphologies, crystallite sizes, chemical compositions, and oxygen isotope disequilibrium “vital effects”^2,26,29,30^. In particular, different orders of foraminifera have different fundamental ultrastructural nanocrystallite shapes and sizes. The nanocrystallites that compose the tests of hyaline foraminifera (order Rotaliida), which is the order most used in recent paleoclimate reconstructions, are subspherical and 50 nm in diameter, whereas Lageniida tests are composed of large >1-μm hollowed single-crystal fibers, and Millioida tests are composed of thin ~400-nm long needle-shaped nanocrystallites^30,31^. Altogether, the properties of foraminifera calcitic tests (and of most other biogenic carbonates) are radically different from the classical crystal properties of abiotic carbonates. Whereas surface reaction(s) and diffusion might be considered negligible at low temperatures in large abiotic minerals, this paradigm may not be valid for biogenic carbonates^12,17^, which feature pathways throughout their ultrastructure that could permit rapid fluid penetration and reactive surface-areas many orders of magnitudes greater than equivalently sized abiotic minerals.

This study examines the susceptibility of calcitic tests of *Ammonia* sp., a genus of benthic hyaline foraminifera from the order Rotaliida, to mineral-fluid oxygen isotope exchange in ^18^O-enriched artificial seawaters (ASWs) at chemical equilibrium with calcite, at low temperatures and over short time scales (days to weeks). The use of ^18^O-enriched fluids permits the rapid quantification and monitoring of changes in isotope compositions at low temperatures, which would otherwise be impossible to detect on experimental time scales. Maintaining elemental chemical equilibrium during these incubations allowed the preservation of the original ultrastructures of the foraminifera tests during the experiments. By focusing on foraminifera, we target organisms that preserve the largest range of seawater temperatures in the paleoclimatic record. Although the experiments presented here were conducted on only one order of foraminifera, the processes, the results, and their implications should be generally applicable to the many organisms that produce carbonate biominerals composed of the same fundamental ultrastructural units^19^ (i.e., subspherical nanocrystallites) as Rotaliid foraminifera.

## Results and discussion

### Ultrastructures of calcitic foraminifera tests

**Fig. 1:**
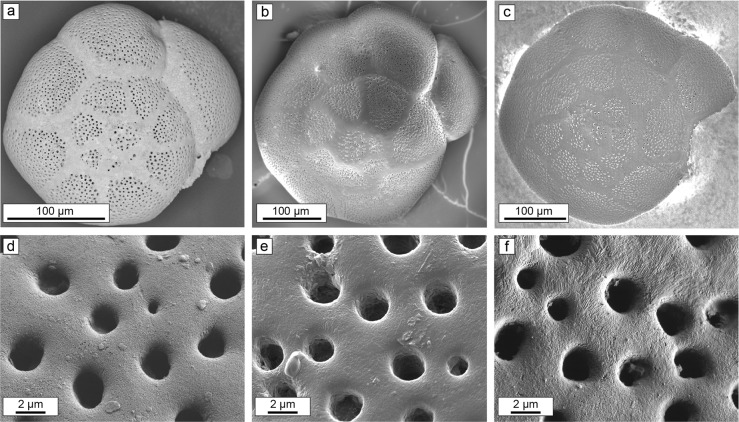
Scanning electron microscope (SEM) images of foraminifera test morphology and respective surface textures prior to and after isotopic exchange experiments. **a** Visually pristine methanol-cleaned foraminifera test prior to incubation. **b** Image of foraminifera test incubated in artificial seawater (ASW) at 90 °C for 54 days with an ^18^O-enrichment at 1000‰ VSMOW. **c** Foraminifera test incubated in Na_2_CO_3_ buffered Milli-Q at 190 °C for 40 days with a ^18^O-enrichment at 1000‰ VSMOW. **d**–**f** Corresponding high-resolution SEM images of panels **a**–**c**, respectively. Note the absence of any visible textural changes in **b** and **c** compared with the pristine un-reacted foraminifera test of image **a**. An extensive set of comparison images between incubated and pristine foraminifera made under the same experimental parameters can be found in the supplementary information of Cisneros-Lazaro et al.^12^.

This study used *Ammonia* sp., a genus of calcitic benthic foraminifera of the order Rotaliida, which is the order of foraminifera most commonly used for paleoseawater temperature reconstructions in the studies examined in this manuscript, which constitute the largest collection of global seawater temperature reconstructions from foraminifera oxygen isotope compositions dating back roughly 120 My^32–37^. Rotaliida foraminifera consist of benthic and planktonic calcitic tests that are multi-chambered, spiraled and perforated by pores. At the nanoscale, the fundamental building blocks of their tests are subspherical nanocrystallites 10–150 nm in diameter, similar to those in most other known biocarbonates^19,30,31,38^. Prior to incubation (for details cf. the Methods section), un-reacted foraminifera tests appeared “glassy” when viewed with an optical microscope. Imaged with a scanning electron microscope (SEM), the pores appeared quasi-circular when viewed down their long axis, and the external surfaces of the foraminifera were smooth; note that some foraminifera tests naturally show signs of minor surface etching and irregular pores (Fig. 1). After incubation the tests still appeared “glassy” when viewed with the optical microscope and remained visually indistinguishable from their un-reacted “pristine” counterparts when observed by SEM (Fig. 1). Occasionally, some pores did show signs of dissolution, however, as noted above, irregular pores are also common features of pristine foraminifera tests. Scanning electron images of nanocrystallites indicates that nanocrystallite shapes and sizes did not change after reaction and no indicators of precipitation or overgrowths were evident at any scale (Fig. 1). In addition, foraminifera tests incubated for different periods of time or at different temperatures exhibited no systematic textural differences.

### Rapid foraminiferal test ^18^O-enrichment

**Fig. 2:**
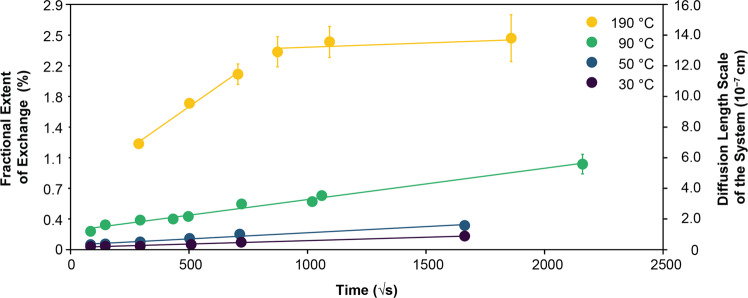
The fraction (*F*) of foraminifera test calcite isotopically equilibrated with artificial seawater enriched in ^18^O to 1000‰ VSMOW plotted against the square root of time for experimental temperatures between 30 and 190 °C. Values on the left axis measure the fractional change in the bulk oxygen isotope ratio of the foraminifera tests as they evolve toward the equilibrium isotope value of the system. The right axis shows the diffusive length scales of the system (*L*), which are calculated by normalizing the product of the number of moles of oxygen exchange and the molar volume, to the aliquot-specific surface area. The diffusion constant for each temperature can be obtained by squaring the slope of the *L* regression line. Note the change in the kinetic regime at 190 °C at an *F* value of 2.4% and *L* of 1.3 × 10^−6^ cm after 8 days. Error bars are typically smaller than the symbol size, but when visible, they correspond to one standard deviation of the variability of the isotopic measurements of the aliquot.

The reaction of foraminifera tests with aqueous fluids at saturation with respect to calcite resulted in easily measurable fluid–mineral oxygen isotope exchange (Supplementary Table 1 and Fig. 2). For all experiments, the oxygen isotope exchange rates correlated positively with temperature and, within individual incubations, gradually decreased with time, producing a linear relationship between the amount of exchange and the square root of incubation time. After 54 days at 90 °C, over 1% of all the oxygen in a foraminifera test was exchanged with the surrounding pore fluid without any discernable textural modification. Of note, exchange rates were only slightly lower for oxidatively cleaned foraminifera compared to non-oxidatively cleaned foraminifera, and aliquots with different fluid:foraminifera test weight ratios showed the same amount of isotope exchange for the same experimental times (Supplementary Table 1).

At an experimental temperature of 190 °C, the oxygen isotope exchange exhibited two kinetic regimes. After 8 days of incubation that resulted in an exchange of about 2.4% of the oxygen in the foraminifera test, the oxygen isotope exchange rate decreased and continued at a much lower rate (Fig. 2). At lower experimental temperatures, a similar shift to a lower rate of isotopic exchange was not noted.

### Mechanism for rapid ^18^O exchange in foraminifera

Given that no textural modifications were observed, e.g., no substantial secondary calcite precipitation or dissolution occurred, the measured low-temperature O-isotope exchange might be attributed to one or several of six processes: adsorption, preferential isotope uptake by organic molecules, precipitation of nanoparticles, pseudomorphic dissolution-precipitation, lattice diffusion, and/or grain-boundary diffusion^13,14,17^. However, any process(es) invoked must be able to explain the following three observations: (1) exchange rates increasing with increasing temperature, (2) exchange rates decreasing with time, and (3) a sudden decrease in exchange rates after an exchange of ~2.4% of all the oxygen in a foraminifera test incubated at 190 °C (Fig. 2).

Here, adsorption can be disregarded as the principal process behind the isotope exchange for several reasons. In our analyses, the number of atoms of oxygen exchanged exceeds the number of oxygen atoms at all sorption sites available on a surface monolayer of calcite on a foraminifera test at any assumed specific surface area between 0.086 and 2 m^2^/g for all analyses except for one data point after 2 h at 30 °C. Although up to 6 monolayers may be accessible for adsorption on a calcite surface^39^, adsorption would still not account for any of the analyses above 90 °C, and with an assumed specific surface area of 0.086 m^2^/g the number of exchanged atoms would still exceed the number of oxygen atoms at adsorption sites by 2–50 times in all but three analyses at 30 °C. Furthermore, adsorption cannot account for the sudden decrease in exchange rates after 190 °C and 8 days unless 2.4% of all the oxygen atoms were available for adsorption, which is an unreasonable adsorption thickness equivalent to 15–330 monolayers (7.5–150 nm) of calcite depending on the surface area. It follows that although we cannot preclude some adsorption occurring during our experimental timeframes, adsorption by itself cannot account for the isotope exchange data presented here.

Likewise, isotope uptake by organic matter cannot account for the isotope exchange seen in our analyses. Oxidative cleaned foraminifera tests, which have had their intracrystalline organics removed, were slightly less enriched (0.27–0.40% exchange) than non-oxidatively cleaned tests (0.35–0.54% exchange) that were incubated for the same amount of time (Supplementary Table 1). This suggests that some organic molecules appear to influence the isotopic enrichment, either by the direct binding of ^18^O to organic molecules, or through some sort of mechanism related to the degradation of organic molecules during seawater incubation. Despite this, the large isotope enrichment in the oxidatively cleaned tests indicates that a large amount of isotope enrichment still occurs in foraminifera that have had their intracrystalline organic frameworks removed. Suppose isotope enrichment occurred exclusively through the binding of ^18^O with organic molecules present in the foraminifera tests, little to no change in the isotope composition of the oxidatively cleaned tests after incubation would be expected. Instead, the amount of isotope exchange continuously increases with time, just like in the non-oxidatively cleaned aliquots (Fig. 2). In addition, ultra-high-resolution NanoSIMS imaging of foraminifera tests incubated in similar seawater analogues has demonstrated that isotope enrichment occurs ubiquitously in foraminifera tests, including in regions with relatively low abundance of organic molecules^12^. Thus, organic molecules appear to play some role in the process behind isotope exchange, but they do not chiefly account for the isotope enrichment of the tests during our experiments.

The precipitation of nanocrystallites, smaller than what could be observed with the SEM, can be ruled out by the results of the variable water:rock experiments (Supplementary Table 1). If the precipitation was driven by the supersaturation of calcite in the seawater solution, solutions with higher water:rock (W:R) ratios would precipitate more nanocrystallites than those with smaller W:R ratios and the resulting foraminifera tests would have higher δ^18^O values. From Supplementary Table 1, this is not the case and variations in the W:R do not correspond with variations in the δ^18^O values of the resulting foraminifera tests.

Pseudomorphic dissolution-precipitation has been previously invoked as a process capable of altering mineral isotope compositions in the absence of any ultrastructural alteration^13,18^, even in foraminifera tests^14^. Although it can explain higher exchange rates at higher temperatures, at a constant temperature and at chemical equilibrium, calculated exchange rates will not decrease with time, unless they are coupled with changes in the mineral surface area. In pseudomorphic dissolution-precipitation reactions, a decrease in the mineral surface area causes a proportional decrease in the mineral dissolution rate^40,41^ and—as a corollary—to the mineral precipitation rate, which is equal to the dissolution rate in pseudomorphic reactions^18,42^. As an example, at 90 °C, a three order of magnitude decrease in the mineral surface area would be required to explain the decrease in isotope exchange rates over 54 days. A three-order magnitude decrease in the surface area with an initial specific surface area of 2 m^2^/g would be equivalent to a surficial transformation of a typical biogenic carbonate to the smooth surface of an Iceland spar^43^. Scanning electron images of the foraminifera tests (Fig. 1) show that no such textural transformation took place during seawater incubation.

In addition, there is no reason why the rate of pseudomorphic dissolution-precipitation would suddenly dramatically reduce after the exchange of 2.4% of the foraminifera test unless isotope equilibrium was suddenly reached in the seawater/foraminifera test system. However, this did not occur in our experiments, where the fractional change in an isotope ratio (*F*) of the foraminifera tests is far below 100% (Supplementary Table 1) and thus the tests remained in isotope disequilibrium with their surrounding fluids during all experimental timeframes. These two points preclude pseudomorphic dissolution-precipitation as the process behind the isotopic exchange for these foraminifera tests.

The effects of lattice diffusion in our experiments can be modeled using the solution of the diffusion equation of diffusion into a semi-infinite medium of plane-sheet geometry^44^, with a homogeneous initial oxygen isotope composition (30.68‰) and a fixed surface isotope composition (1000‰) following:

$$\frac{{C}_{s}-{C}_{(x,t)}}{{C}_{s}-{C}_{{{{{{\rm{i}}}}}}}}={erf}\left(\frac{x}{2\sqrt{{Dt}}}\right)$$

where *C*_*s*_, *C*_(*x*,*t*)_, and *C*_*i*_ correspond respectively to the isotopic composition of the fluid, the isotopic composition at a point (*x*) in the nanocrystallite at a certain time (*t*), and the initial isotope composition of the nanocrystallite, and *D* is the oxygen diffusion coefficient, which ranges from 10^−31^ to 10^−38^ m^2^s^−1^ at 30 °C and from 10^−27^ to 10^−28^ m^2^s^−1^ at 190 °C^17,45,46^ in biogenic and abiogenic calcite. By integrating the oxygen isotope composition across a nanocrystallite 65 nm in diameter and dividing it by the diameter of the nanocrystallite, the estimated isotope composition of a nanocrystallite due to lattice diffusion after a certain amount of time can be calculated. However, at the low incubation temperatures and short experimental timeframes of this study, lattice diffusion cannot affect the isotope compositions of foraminifera, regardless of which diffusion coefficient is used. At 190 °C, if the highest calculated diffusion coefficient of Farver^46^, Anderson^45^ and Bernard et al.^17^ is used, lattice diffusion would raise the isotopic composition of the foraminifera by only ~2‰ after 8 days, which is an order of magnitude lower than the measured isotopic value (Supplementary Table 1). Therefore, lattice diffusion is much too slow to account for the rapid and substantial change in oxygen isotope composition observed in our experiments.

Oxygen grain-boundary diffusion remains as the only mechanism that could explain the initial rapid isotope exchange, the decreasing isotope exchange rates with time, the increasing isotope exchange rates with increasing temperature, and the observed change to a lower kinetic rate of exchange after 8 days in incubation experiments run at 190 °C. In the following discussion, the term grain-boundary diffusion is used in reference to the transfer and segregation of oxygen, regardless of its speciation, within crystalline grain boundaries.

### Oxygen grain boundary diffusion in foraminifera

In polycrystalline materials, such as biominerals, grain boundary diffusion is one of the fastest mechanisms allowing diffusive isotope uptake^47^. In abiotic calcite, oxygen grain boundary diffusion has been measured to be up to 6 orders of magnitude faster than oxygen volume diffusion^48^, with an activation energy of oxygen grain boundary diffusion (*Ea*_*GB*_) as low as 127 kJ mol^–1^ for abiotic calcite^49^. Here, fitting our measured oxygen grain boundary diffusion coefficients from *Ammonia* sp. to the Arrhenius relationship gives an *Ea*_*GB*_ of 46 kJ mol^–1^, and an average *D*_0_ of 1.6 × 10^−13^ cm^2^/s^−1^ when using a specific surface area of 2.0 m^2^/g, and an average *D*_0_ of 8.8 × 10^−11^ cm^2^/s^−1^ when using a specific surface area of 0.086 m^2^/g, i.e., grain boundary diffusion rates are substantially faster in biogenic foraminifera carbonate tests than in abiogenic carbonates.

This activation energy of grain boundary diffusion is considerably lower than the measured activation energy of oxygen isotope exchange of any other process in an abiotic calcite^45,46,49–51^, which is likely due to the presence of organic molecules and the polycrystalline ultrastructure of the biominerals. Biogenic carbonates are distinguished from abiogenic crystals by the presence of inter- and intracrystalline organic molecules, which produce anisotropic lattice distortions, longer C–O bond lengths, and voids in the crystal structure^52,53^. These crystalline modifications enhance the probability of atoms to migrate through the crystal lattice or along grain boundaries, and consequently lower the activation energies of isotope exchange processes^54,55^. In addition, some degradation of the organic matter during incubation, or during diagenesis in natural settings, could enhance solution penetration into the foraminifera test ultrastructure and result in greater surface areas for isotope exchange^12^. In aragonitic gastropod shells, this degradation of organic matter along with biogenic polycrystalline ultrastructures have been linked to *Ea* of aragonite–calcite transformations that are 40% lower than abiotic aragonite–calcite transformations^56^. Although the removal of intercrystalline organic matter within the foraminifera tests by oxidative treatment prior to incubation did not substantially modify oxygen isotope compositions in our experiments, these treatments do not remove the minor amount but ubiquitous intercrystalline organic matter within foraminifera tests^28^. It is therefore possible that the remaining intercrystalline organic material may explain the lowered activation energy of grain boundary diffusion.

The change in the slope of the oxygen isotope exchange curve after 8 days at 190 °C suggests a change in the kinetic regime/process after approximately 2.4% of all the oxygen in an *Ammonia* sp. test has been exchanged along its grain boundaries (Fig. 2). Depending on the assumed grain boundary thickness (δ), this suggests that the average diameter of a spherical *Ammonia* sp. nanocrystallite lies between 65 and 115 nm (δ = 0.5–1 nm^54,57^), which is consistent with SEM observations^30,31^. After the equilibration of these grain boundaries with oxygen in the pore fluids, grain boundary diffusion ceases to change the O-isotope value of the foraminifera tests. In this experiment, subsequent O-isotope exchange can thus only be attributed to the effects of the much slower lattice diffusion at 190 °C. The grain boundary diffusion rates obtained at 30, 50, and 90 °C indicate that at these temperatures, a foraminifera test consisting of a grain boundary volume fraction of 2.4% would take only 44, 13, and 2 years, respectively, to isotopically equilibrate the grain boundaries with their surrounding pore fluids. Extrapolating these rates to 0 °C, it would take only about 100 years to isotopically equilibrate the grain boundaries inside a foraminifera test with their surrounding pore fluids via grain boundary diffusion.

### Implications for paleoseawater reconstructions from biocarbonates

The present results provide robust new evidence that even fossil foraminifera tests (and fossil marine biocarbonates in general) appearing ultrastructurally pristine almost certainly have exchanged oxygen isotopes with pore fluids, even at low temperatures and on short time scales. The addition of grain-boundary diffusion to lattice diffusion and dissolution-precipitation as low-temperature processes affecting fossil foraminifera tests in ocean sediments further implies that isotopic compositions of fossil biocarbonates in general are not completely faithful recorders of their original precipitation temperatures and conditions. The present results emphasize the importance of characterizing biocarbonates as isotopic systems composed of two oxygen-isotopic reservoirs: a large lattice-bound oxygen-isotopic reservoir and a smaller but constantly exchanging reservoir of grain-boundary oxygen.

The extent of grain boundary isotope exchange in any biomineral is a function of the time it has spent surrounded by a particular fluid at a given temperature, the size of its nanocrystallites, and the thickness of its grain boundaries. Grain boundary diffusion rates are rapid enough that the grain boundaries of any biomineral will be at isotopic equilibrium with their surrounding pore fluid at normal ocean sediment burial temperatures and time scales of about 100 years. Organism-dependent calcite nanocrystallite diameters range from 20 to 450 nm, with the smallest nanocrystallites of many organisms in the range between 20 and 50 nm^58–61^, but the grain boundary widths of these nanocrystallites have never been measured. In abiotic minerals, grain boundary widths are considered to be in the range of 0.5–3 nm, with most studies setting their width between 0.5 and 1 nm^54,57^. Assuming a similar grain boundary width for biogenic carbonates leads to grain boundaries contributing for 2–9% of the total volume of biocarbonates in foraminifera tests. In our experiments, a grain boundary volume fraction of 2.4% is already sufficient to introduce a bias in the estimation of the water temperature based on oxygen isotope compositions.

**Fig. 3:**
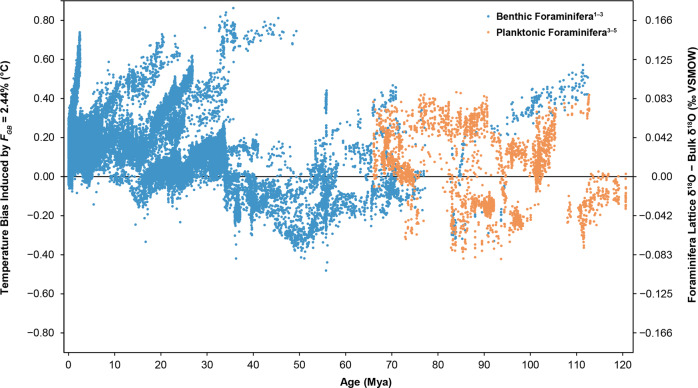
Temperature biases due to grain boundary diffusion in fossil benthic and planktonic foraminifera tests. The left *y*-axis shows the temperature bias induced by grain boundary diffusion on existing seawater paleotemperature reconstructions from bulk test oxygen isotopic compositions. Positive/negative values indicate that grain boundary diffusion has caused existing paleotemperature estimates to be too high/low and should therefore be subtracted by the indicated individual temperature correction. The parameters used to calculate these values are as follows: grain boundary fraction (*F*_*GB*_ = 2.4%), seafloor temperature = 2.67 °C^63^, seawater δ^18^O_VSMOW_ = 0‰, and a geothermal gradient = 0.053 °C/m. In boreholes with known geothermal gradients and seafloor temperatures, the known parameters were used in the calculations. Temperatures were calculated by multiplying the foraminifera δ^18^O_Lattice−_δ^18^O_Bulk_, shown on the right *y*-axis, by 4.81 °C/‰. The steep increase in benthic temperature biases at around 5 Mya is from foraminifera from Site 677, where the geothermal gradient is 0.208 °C/m^63^. Benthic foraminifera are from (1) Zachos et al. (2008)^32^, (2) Borrelli and Katz (2015)^33^, (3) Cramer et al. (2009)^34^, and (4) Huber et al. (2018)^35^. Planktonic foraminifera are from (4) Huber et al. (2018)^35^, (5) O’Brien et al. (2017)^36^, and (6) O’Connor et al. (2019)^37^.

Regardless of the original precipitation temperature and any post-diagenetic alteration, the bulk δ^18^O value of a foraminifera test has inevitably been modified by the changes in its grain boundary δ^18^O value from being surrounded by sediment pore fluids with modern δ^18^O values at ambient burial temperatures during the last 100 years. To eventually correct this bias in the foraminifera paleotemperature record, we estimated the effect of oxygen grain boundary diffusion on the isotopic composition of 38,470 fossil benthic and planktonic foraminifera tests obtained from 89 boreholes and covering a timespan from the present to the early Cretaceous^32–37,62^. The use of parameters estimated for *Ammonia* sp. can be seen as conservative, given that previous work demonstrated that isotope exchange is slower for *Ammonia* sp. than for other benthic Rotaliida foraminifera species due to differences in test ultrastructure and the relative abundances of organic matter^12^. Figure 3 shows the calculated corrections (due to grain boundary diffusion alone) of the seawater paleotemperatures reconstructed from the oxygen isotope compositions of the 38,470 foraminifera test analyses in the current sediment record^62^, using geothermal gradients from borehole data when available and otherwise using an average geothermal gradient of 0.053 °C/m^63^ and an average modern-day seawater δ^18^O_VSMOW_ value of 0‰^64^. Oxygen isotope exchange between the grain boundaries and the pore fluid will not alter the isotope composition of the fluid because the average porosity of sediment at the average burial depth of the 38,470 foraminifera tests (177 m) is around 70%^65^, and the molar ratio of oxygen in the pore fluids to oxygen in the grain boundaries exceed a ratio of 60:1, i.e., systems isotopically buffered by the pore fluids. Note that the potential re-equilibration due to solid-state diffusion discussed by Bernard et al.^17^ is not taken into account here, nor are any other effects of diagenesis. Negative values in Fig. 3 indicate that oxygen grain boundary diffusion has caused the reconstructed seawater paleotemperature from the measured bulk foraminifera δ^18^O value to be overestimated; positive temperature corrections in Fig. 3 indicate the opposite.

Because the effect of grain boundary diffusion on the fossil foraminifera tests is driven by temperature-dependent isotope disequilibrium between the calcitic tests and the surrounding pore fluids, benthic and planktonic foraminifera will generally be affected differently. Benthic foraminifera already live at the coldest temperature in the water column, thus once burial begins, they gradually become subjected to hotter temperatures and the isotopic disequilibrium increases with burial depth. As such, without correction for grain boundary diffusion, the reconstructed deep-ocean water temperatures from benthic foraminifera tests will tend to overestimate the original deep-ocean seawater temperatures. Planktonic tests, instead, are generally precipitated in seawater temperatures higher than those at the seafloor. In that case, the greatest isotope disequilibrium between pore fluids and planktonic tests occurs at the moment the organisms die, sink, and settle on the cooler sediments. Once burial begins and pore fluid temperatures increase, the isotopic disequilibrium between the test and the pore fluids decreases, resulting in smaller temperature biases from grain boundary diffusion. Therefore, depending on the burial temperature and the water temperature originally experienced by the planktonic foraminifera, estimated paleotemperatures from planktonic foraminifera tests can be lower, equal to, or higher than their original seawater temperatures (Fig. 3). Consequently, the most biased benthic foraminifera paleotemperature records are from tests retrieved from warm and deeply-buried Oligocene sediments, whereas the most biased planktonic foraminifera tests are those that come from relatively low-temperature shallow-buried Late Cretaceous sediments.

Figure 3 shows many benthic and planktonic foraminifera that do not follow these generalizations. These generalizations will hold so long as seawater temperatures decrease with depth in the water column, pore fluid temperatures increase with burial depth, no uplift/erosion has occurred, seafloor temperatures have not changed with time, and no recrystallization has occurred. For many sediments hosting fossil foraminifera tests, these conditions have not been maintained. In sediments that have been eroded or uplifted, foraminifera tests will be subject to lower burial temperatures than if they had been continuously buried. In other cases, seafloor temperatures appear to have changed throughout time and so have the resulting burial and ambient temperatures, which causes some benthic foraminifera tests to be biased toward higher or lower temperatures depending on their burial depth. In the case of recrystallized foraminifera tests, these tests are likely somewhat protected from the effects of grain boundary diffusion. Recrystallized tests show increased crystallite sizes, overall surface area decreases, and smaller grain boundary volume fractions^2,8^. As a corollary, the more recrystallized a foraminifera test is, the less pronounced are the effects of grain boundary diffusion. In most cases, since grain boundary diffusion occurs after recrystallization, grain boundary diffusion in recrystallized tests should nearly always move temperatures toward hotter values, except in the cases where seafloor temperatures have changed dramatically over time or if the sediments have undergone erosion/uplift and ambient temperatures have decreased.

Temperature biases induced by grain boundary diffusion add another layer of uncertainty to paleotemperature reconstructions. Initially, any temperature calculation uses the δ^18^O value of a foraminifera test, which is composed of three volume fractions: (1) the unaltered original pristine δ^18^O fraction, (2) a diagenetically altered δ^18^O fraction and (3) the grain boundary volume fraction. Thus, the error in a paleotemperature from a foraminifera test is a composite of numerous unavoidable sources of uncertainty ranging from measurement errors, biogenic factors, estimations of ancient water parameters, diagenetic histories, interspecies calibrations, organism and taphonomic sampling biases, and the bulk averaging of data from different sites through time^2,34,66^.

Unlike other uncertainties related to estimates of ancient ocean temperatures, such as the δ^18^O_seawater_ value and diagenetic changes, the effect of grain boundary diffusion on a test can be corrected by three measurable properties: the pore fluid δ^18^O value, the ambient burial temperature when the test was collected, and the measured grain boundary volume fraction. The first two properties are often available from borehole technical reports and the grain boundary volume fraction can be measured following the techniques introduced in this manuscript. Provided this correction is applied, the resulting δ^18^O value is still not that of the original foraminifera test δ^18^O value, which still is a mixture of the original test δ^18^O value and δ^18^O value of diagenetically produced carbonate. It is from this mixed carbonate material that a paleoseawater temperature is currently estimated. Although the effects of grain boundary diffusion may be less than the implied errors in paleotemperature reconstructions, grain boundary diffusion causes a notable but easily correctable offset to foraminifera δ^18^O values.

An additional effect of such high grain boundary diffusion coefficients (Table 1) is that the effective lattice diffusion coefficient for biocarbonates at low temperatures is substantially increased by the fast penetration of external oxygen throughout their polycrystalline structure^67,68^. In addition, if the rate of oxygen isotope exchange measured at 190 °C after 8 days (Fig. 2) is attributable to lattice diffusion, the oxygen lattice diffusion coefficient at 190 °C is (4.4 ± 2.6) × 10^–25^ m^2^s^–1^, which is 3 orders of magnitude faster than the extrapolated diffusion coefficient from abiotic calcite at 190 °C^46^, but consistent with Bernard et al.^17^, who showed that oxygen diffusion coefficients derived from planktonic foraminifera tests are much larger than in abiotic minerals^17^. As previously suggested by Bernard et al.^17^, this implies that, given sufficient time, low-temperature lattice diffusion may—in addition to grain boundary diffusion—appreciably alter biomineral oxygen isotope compositions and bias paleotemperature reconstructions.

**Table 1:** Calculated grain boundary diffusion coefficients.

Temp. (°C)	*L*_slope_ (cm•s^−0.5^) (2.0 m^2^/g)	*L*_slope_ (cm•s^−0.5^) (0.086 m^2^/g)	*D*_*GB*_ (cm^2^ s^-1^) (2.0 m^2^/g)	*D*_*GB*_ (cm^2^ s^-1^) (0.086 m^2^/g)	ln(*D*_*GB*_) (2.0 m^2^/g)
30	(4.451 ± 0.243) × 10^−11^	(1.035 ± 0.057) × 10^−9^	(1.981 ± 0.108) × 10^−21^	(1.072 ± 0.059) × 10^−18^	−47.671 ± 0.055
50	(7.933 ± 0.636) × 10^−11^	(1.845 ± 0.148) × 10^−9^	(6.293 ± 0.504) × 10^−21^	(3.404 ± 0.273) × 10^−18^	−46.515 ± 0.080
90	(1.998 ± 0.092) × 10^−10^	(4.647 ± 0.215) × 10^−9^	(3.994 ± 0.184) × 10^−20^	(2.160 ± 0.100) × 10^−17^	−44.667 ± 0.046
190	(1.040 ± 0.083) × 10^−9^	(2.419 ± 0.194) × 10^−8^	(1.082 ± 0.087) × 10^−18^	(5.851 ± 0.469) × 10^−16^	−41.368 ± 0.080

Grain boundary diffusion coefficients (*D*_*GB*_) are calculated from the slopes of the diffusive length scale of the system (*L*_*slope*_) using a specific surface area of 0.086^14^ and 2 m^2^/g^75^. Since the activation energy is calculated from the slope of the ln(*D*_*GB*_) values, either surface area can be used to calculate ln(*D*_*GB*_) without any change to the activation energy.

Despite the high oxygen grain boundary and lattice diffusion rates reported in this study, foraminifera trace element compositions should be relatively robust to changes through lattice and grain boundary diffusion. Low-temperature extrapolation of diffusion coefficients of metals in calcite, which are orders of magnitude smaller than for oxygen at the same temperature, imply that at ambient temperatures, most metal ions in crystal lattices are immobile^69^. The Sr, Ca, and Mg lattice diffusion coefficients in calcite^49,70,71^ extrapolated to 25 °C are, respectively, 10^–36^, 10^–68^, and 10^–54^ m^2^s^–1^, i.e., many orders of magnitude smaller than the reported oxygen values. This, coupled with the much smaller volume of grain boundaries compared with the matrix, suggests that lattice and grain boundary diffusion of metals should not influence trace element compositions of foraminifera tests.

In conclusion, rapid oxygen grain boundary diffusion in biocarbonates can proceed without imparting visually perceptible changes to (ultra)structure and result in biased seawater paleotemperature reconstructions. The extent of this bias will depend on the original isotopic composition of the biominerals, their biocarbonate nanostructure, the δ^18^O of the porewater, and the temperature at which they have stayed during the last 100 years. In general, the present study suggests that grain boundary diffusion alone will lead to an overestimation of bottom ocean water paleotemperatures and to over- or under-estimation of sea surface paleotemperatures, with the combined effect of a greater difference between bottom-water and sea-surface temperatures during the last 120 My. Although ubiquitous in the paleotemperature record, this diffusional temperature bias can be corrected for by estimating the grain boundary fraction of the biomineral used for the temperature reconstruction. Unfortunately, biominerals have rarely been the focus of diffusional studies. Their characterization with respect to grain boundary widths, nanocrystallite ultrastructures, and diffusion coefficients is however an important next step to accurately correct existing biases in the paleo-proxy record, which naturally also integrates the effects of other overt diagenetic overprints.

## Methods

### Preparation of starting materials

This study used modern, shallow-water benthic foraminifera of the genus *Ammonia* sp., collected from modern tidal sediments on the coast of western France (La Rochelle). Batches of 100–300 *Ammonia* sp. (0.5–3.9 mg) were picked from wet-sieved sediments and then sonicated in methanol and deionized Milli-Q® water, following the standard foraminifera cleaning procedure of Barker et al.^72^. Some batches of foraminifera were also oxidatively cleaned with buffered H_2_O_2_ + NaOH solution after Barker et al.^72^. Following the cleaning step, the foraminifera were desiccated at 37 °C for 24 h.

Aliquots of foraminifera were placed in flame-sealed borosilicate tubes or sealed in arc-welded gold capsules, filled to ~70% volume percentage with solution with the remaining headspace as air, and then placed in ovens at temperatures between 30 and 190 °C for 2–1296 h (54 days). At temperatures between 30 and 90 °C, foraminifera tests were reacted with about 60 mg of synthetic seawater solutions saturated with respect to calcite (Saturation Index (SI) = 1), whereas at 190 °C, foraminifera were reacted with about 60 mg of deionized Milli-Q® water, buffered to a pH of 8.83 by the addition of Na_2_CO_3_ (Table 2). A 0.01 M Na_2_CO_3_ solution was used at 190 °C instead of a seawater solution because seawater solutions with numerous dissolved components increase the risk of adverse chemical reactions at high temperatures, but more importantly, they are more undersaturated with respect to calcite at room temperature than an equivalent 0.01 M Na_2_CO_3_ solution. Using the geochemical computer program PHREEQC^73^, an ASW solution, chemically equilibrated with calcite at 190 °C, is undersaturated with respect to calcite at room temperature (SI = −1.16) and given enough time would dissolve ~20 μg of calcite in 50 μL of solution. The precipitation of 20 μg of calcite in an aliquot of 1500 μg of calcite at 190 °C would produce at calcite with an isotopic composition ~13‰ higher than the original isotope ratio before any other isotope exchange, which would be an unacceptable amount of precipitation. Instead, a 0.01 M Na_2_CO_3_ solution, which is undersaturated with respect to calcite at 190 °C, would only dissolve 0.47 μg of calcite at room temperature and only 0.03 μg of calcite at 190 °C to reach chemical equilibrium. The precipitation of 0.44 μg of calcite at 190 °C would lead to an enrichment of 0.28‰ of the calcite test, which we acknowledge is the maximum amount of isotope enrichment from precipitation that may be present in our measured values. At 190 °C, the fluid in the gold-capsule reached a maximum pressure of approximately 16 bar but remained as a single-phase fluid, as opposed to superheated vapor. To verify if a small residual chemical disequilibrium had an effect on the observed isotope exchange, we conducted experiments at the same temperature and the same experimental time frame but with different solution:calcite ratios. Changing the amount of fluid from about 20 to 300 mg had no effect on the observed isotope exchange (Supplementary Table 1), indicating that isotope exchange is not influenced by the small residual disequilibrium, which can result in small amounts of dissolution during the experiment run times. Speciation was calculated using the aqueous geochemistry software PHREEQC and the Davies activity equation^73^.

**Table 2:** Water chemistry of reaction fluids.

Temperature (°C)	Na (mol/L)	Ca (mol/L)	Cl (mol/L)	Mg (mol/L)	pH
30	0.42490	0.00041	0.52890	0.05289	9.60
50	0.42960	0.00058	0.53910	0.05562	9.14
90	0.52460	0.00100	0.61740	0.04702	8.27
190	0.01000	–	–	–	8.83

After the reaction, the reaction containers were quenched in cool water for less than a minute and cleaned with Milli-Q® water and technical-grade ethanol. The foraminifera tests were then desiccated in an oven at 50 °C for 24 h, followed by at least 24 h in a vacuum desiccator to remove adsorbed water. Student’s *t*-test performed on two aliquots of a cooked and oven-dried batch of foraminifera tests, where one aliquot was vacuum desiccated prior to measurement and the other aliquot was not, indicates that inclusion or omission of vacuum desiccation does not affect the isotopic composition of the measured foraminifera tests (*t*(21) = −0.530, *p* = 0.6018). After desiccation, foraminifera were examined first under optical microscopy and then with a Zeiss Gemini 500 SEM for signs of recrystallization, dissolution, or precipitation.

Foraminifera oxygen isotope ratios were analyzed at the University of Lausanne using a Finnigan Delta V Advantage mass spectrometer coupled to a GasBench II by reaction with phosphoric acid at 70 °C. Each analysis consists of an average of 10–30 aliquots of 60 µg of foraminifera (~7 foraminifera). Isotopic ratios were corrected online for phosphoric acid fractionation by a Carrara marble internal standard. Isotope measurements are reported in the δ-notation in parts per thousand (‰) relative to Vienna Standard Mean Water (VSMOW). Uncleaned, methanol-cleaned, and methanol + oxidatively cleaned foraminifera had average δ^18^O_VSMOW_ values of 30.68 ± 0.42‰ (1σ) (*n* = 29), 30.68 ± 0.46‰ (1σ) (*n* = 137), and 30.96 ± 0.43‰ (1σ) (*n* = 26) respectively. Methanol cleaning did not affect the oxygen isotopic composition of the foraminifera (*t*(194) = 0.022, *p* = 0.982), whereas oxidative cleaning slightly increased oxygen isotopic compositions (*t*(53) = −2.417, *p* = 0.0191), which are both consistent with previous studies of cleaning procedures on foraminifera tests^72,74^. All experimental fluids were enriched in ^18^O to δ^18^O_VSMOW_ = 1000 ± 1‰ by weighing a small amount (<10 mg) of ^18^O-enriched water (97 atom% ^18^O) on a microbalance with 0.1 mg precision. Then the appropriate weight of non-enriched ASW solution (−12‰ δ^18^O_VSMOW_) was calculated using the fractional isotope abundances of each fluid to produce a solution with a δ^18^O_VSMOW_ of 1000‰. Analytical precision for foraminifera oxygen isotope analysis was approximately ±0.1‰ based on replicate standard analyses.

The surface area of foraminifera tests is a difficult parameter to estimate. Brunauer–Emmett–Teller surface area estimates of *Ammonia* tests could not be conducted because tens of thousands of tests are required for a single surface area measurement. Previous specific surface area estimates for foraminifera tests range from 0.086 to 3.5 m^2^/g^75–77^. For our purposes, we use the foraminifera test-specific surface area estimates of Chanda et al.^14^ and Honjo and Erez^75^ of 0.086 and 2 m^2^/g, respectively, and we consider the specific surface area to be constant with time, as discussed in the results.

### Isotope exchange calculations

The fractional change in an isotope ratio of a mineral as it evolves toward the equilibrium value of the system is:

1 $$F(t)=\frac{{{{{{{\rm{\delta }}}}}}}_{t}-{{{{{{\rm{\delta }}}}}}}_{i}}{{{{{{{\rm{\delta }}}}}}}_{{eq}}-{{{{{{\rm{\delta }}}}}}}_{i}}$$

where *δ*_*i*_, *δ*_*eq*_, and *δ*_*t*_ are the isotopic compositions of the mineral initially, at equilibrium, and in between at time = *t*, respectively^13,15,78^. However, *F* does not indicate the number of atoms exchanged at any given time^13,15^. The number of atoms exchanged between the foraminifera test calcite and experimental fluid is given by the mass balance equation:

2 $${{{{{{\rm{\delta }}}}}}}_{{sys}}\times {N}_{{sys}}={{{{{{\rm{\delta }}}}}}}_{{aq}}^{t}\times {N}_{{aq}}+{{{{{{\rm{\delta }}}}}}}_{{cal}}^{t}\times {N}_{{cal}}$$

where *δ*_*sys*_, *δ*^*t*^_*aq*_, and *δ*^*t*^_*cal*_ are the isotopic compositions of the entire system, the fluid, and the mineral at time = *t*, respectively. The total moles of oxygen in the system, fluid, and calcite are represented by *N*_*sys*_, *N*_*aq*_, and *N*_*cal*_, respectively. At time = 0, *δ*^*t*^_*aq*_, and *δ*^*t*^_*cal*_ are the initial oxygen isotope compositions of the fluid and calcite prior to any exchange. Due to the high fluid:mineral molar oxygen ratios and the low percentage of exchange, the oxygen isotopic composition of the water (*δ*^*t*^_*aq*_) remains effectively constant during the run time and always equals the initial oxygen isotope ratio of the fluid.

At any time, the oxygen isotopic composition of a calcitic foraminifera test consists of the isotope ratios of the unexchanged oxygen atoms and exchanged oxygen atoms:

3 $${{{{{{\rm{\delta }}}}}}}_{{cal}}^{t}\times {N}_{{cal}}={{{{{{\rm{\delta }}}}}}}_{{cal}}^{i}\times {N}_{{cal}}^{{not}\,{ex},\,t}+{{{{{{\rm{\delta }}}}}}}_{{cal}}^{{ex},t}\times {N}_{{cal}}^{{ex},\,t}$$

where *δ*^*i*^_*cal*_ is the initial oxygen isotopic composition of the foraminifera test and *δ*^*ex, t*^_*cal*_ is the oxygen isotopic composition of the exchanged calcite at time = *t*. The numbers of unexchanged and exchanged oxygen atoms in calcite are represented by *N*^*not ex, t*^_*cal*_ and *N*^*ex,t*^_*cal*_, respectively, and *N*^*not ex, t*^_*cal*_ + *N*^*ex, t*^_*cal*_ = *N*_*cal*._ The isotopic composition of the exchanged fraction of calcite is controlled by the isotopic composition of the fluid and the oxygen isotope fractionation at the reaction temperature:

4 $${{{{{{\rm{\delta }}}}}}}_{{cal}}^{{ex},\,t}={\alpha }_{{cal}-{water}}\left(1000+{{{{{{\rm{\delta }}}}}}}_{{aq}}^{{{{{{\rm{t}}}}}}}\right)-1000$$

where *α*_*cal-water*_ is the fractionation factor between water at the experimental temperature and a calcite in equilibrium at that temperature, and *δ*^*t*^_*aq*_ equals 1000‰ or when converted to the isotopic fractional abundance of ^18^O it equals 0.0039944.

By substituting Eqs. 3 and 4 into Eq. 2 and solving for *N*^*ex,t*^_*cal*_ yields:

5 $${N}_{{cal}}^{{ex},t}=\frac{{N}_{{cal}}\times ({{{{{{\rm{\delta }}}}}}}_{{cal}}^{{{{{{\rm{t}}}}}}}-{{{{{{\rm{\delta }}}}}}}_{{cal}}^{{{{{{\rm{i}}}}}}})}{({\alpha }_{{cal}-{water}}\left(1000+{{{{{{\rm{\delta }}}}}}}_{{aq}}^{{{{{{\rm{t}}}}}}}\right)-1000-{{{{{{\rm{\delta }}}}}}}_{{cal}}^{{{{{{\rm{i}}}}}}})}$$

and by rearranging Eq. 5, the fractional extent of exchange (*N*^*ex, t*^_*cal*_/*N*_*cal*_) can be calculated:

6 $$F(t)=\frac{({{{{{{\rm{\delta }}}}}}}_{{cal}}^{{{{{{\rm{t}}}}}}}-{{{{{{\rm{\delta }}}}}}}_{{cal}}^{{{{{{\rm{i}}}}}}})}{({\alpha }_{{cal}-{wat}{er}}\left(1000+{{{{{{\rm{\delta }}}}}}}_{{aq}}^{{{{{{\rm{t}}}}}}}\right)-1000-{{{{{{\rm{\delta }}}}}}}_{{cal}}^{{{{{{\rm{i}}}}}}})}$$

which is nearly identical to Eq. 1 and provides a method of assessing the fractional extent of oxygen isotope exchange relative to isotopic equilibrium.

### Grain boundary diffusion calculations

To calculate grain boundary diffusion coefficients, the amount of oxygen exchanged in each aliquot was converted to a diffusive length scale of the system (*L*) in centimeters by multiplying the number of moles of oxygen exchanged (*N*^*ex, t*^_*cal*_) by the molar volume of calcite (*V*_*cal*_ = 36.918 cm^3^/mol)^79^, and normalizing to the aliquot surface area, i.e., mass of the aliquot in grams (*M*) multiplied by the foraminifera-specific surface area of 0.086 or 2 m^2^/g (σ):

7 $${L}_{\left(t\right)}={V}_{{cal}}\times {N}_{{cal}}^{{ex},t}/(M\times {{{{{\rm{\sigma }}}}}})$$

These values were plotted with respect to the square root of time and the slope of the linear regression line (*L*_*slope*_ in cm•s^−0.5^) for each temperature was squared to calculate a grain boundary diffusion coefficient:

8 $${{{{{{\rm{Diffusion}}}}}}}\,{{{{{{\rm{coefficient}}}}}}}\left(\frac{{{{{{{{\rm{cm}}}}}}}}^{2}}{{{{{{\rm{s}}}}}}}\right)={L}_{{slope}}^{2}$$

The activation energy of grain boundary diffusion was calculated by taking the slope of the natural logarithm of all diffusion coefficients, plotted as a function of the reciprocal corresponding temperatures. The use of different specific surface areas does not change the calculation of the activation energy. All calculated diffusion coefficients and their errors are listed in Table 1.

Although diffusion coefficients are only directly calculated from our measured data between 30 and 190 °C, we propose they can be extrapolated accurately to lower temperatures above the freezing point of water. When diffusion coefficients are extrapolated to low temperatures from high-temperature data (>400 °C), they may be under or overestimated due to differences in changes to the mineral/solution phases, pressure differences, mineral thermal expansion/contraction, or changes in oxygen fugacity, in the mineral or solution between high and low temperatures^54,80,81^. However, we determined the low temperature, i.e., 30 and 50 °C, diffusion coefficients in temperature and chemical conditions analogous to the shallow burial setting in a sediment column. In addition, the diffusion coefficients calculated at high temperatures, i.e., 90 and 190 °C, can be precisely calculated from and are consistent with the low-temperature diffusion coefficients, despite any potential difference in the mineral/solution properties between 30 and 190 °C (Supplementary Fig. 1). This implies that the same process is occurring throughout the examined temperature range, otherwise a deviation in the calculated diffusion coefficient would be observed. Since the mineral/solution properties listed above show greater differences between 30 and 190 °C, than we would expect between 2 and 30 °C, we believe that the extrapolation of our data to lower temperatures is reasonable.

### Oxygen sorption sites calculation

The number of atoms of oxygen available for adsorption on a calcite surface monolayer of any aliquot can be calculated by multiplying the density of CO_3_ sites on a calcite surface (8.22 × 10^−6^ mol/m^2^)^82^, the aliquot-specific surface area, and a foraminifera-specific surface area between 0.086 and 2 m^2^/g:

9 $${{{{{\rm{O}}}}}}\,{{{{{{\rm{available}}}}}}}\,{{{{{{\rm{for}}}}}}}\,{{{{{{\rm{adsorption}}}}}}}\,({{{{{{\rm{mol}}}}}}})=	 \,3\times {{{{{{\rm{Aliquot}}}}}}}\,{{{{{{\rm{mass}}}}}}}\,\left({{{{{\rm{g}}}}}}\right)\\ 	 \times S.A.\,({{{{{{\rm{m}}}}}}}^{2}/{{{{{\rm{g}}}}}})\times {8.22}^{-6}({{{{{{\rm{mol}}}}}}}/{{{{{{\rm{m}}}}}}}^{2})$$

Using a foraminifera-specific surface area of 2 m^2^/g, the ratio of the moles of oxygen exchanged to the available moles of oxygen on the calcite surface area ranges from 0.23 to 15.06 with 63% of the values above 1, whereas with a surface area of 0.086 m^2^/g the ratio ranges from 5.30 to 350.25.

### Calculating the effect of grain boundary diffusion

The isotopic compositions of planktonic and benthic foraminifera tests (number of individual analyses, *n* = 38470) from 89 boreholes^62^, used for paleoseawater temperature reconstructions from the present to the early Cretaceous^32–37^, were corrected for the effects of grain boundary diffusion. The ambient porewater temperature was calculated by multiplying the sediment depth of each fossil foraminifera test sample by a geothermal gradient of 0.053 °C/m^63^ and adding an average bottom-water temperature of 2.67 °C^63^. In boreholes where the bottom-water temperature and geothermal gradient were known (*n* = 21)^63^, the ambient porewater temperature was calculated using those parameters. Using a grain boundary fraction (*F*_*GB*_) of 2.4%, which is the *F* value at which all grain boundaries have equilibrated oxygen isotopes with the seawater analogue after 8 days in the experiment run at 190 °C, the actual/corrected isotopic composition of the foraminifera test was calculated by:

10 $${\delta }^{18}{{{{{{\rm{O}}}}}}}_{{{{{{{\rm{Lattice}}}}}}}}=\frac{{\delta }^{18}{O}_{{{{{{{\rm{Bulk}}}}}}}}-{\delta }^{18}{{{{{{\rm{O}}}}}}}_{{{{{{{\rm{Grain}}}}}}}\,{{{{{{\rm{boundary}}}}}}}}\times ({F}_{{GB}})}{1-{F}_{{GB}}}$$

where δ^18^O_Lattice_ is the isotope composition of the test corrected for grain boundary diffusion, δ^18^O_Bulk_ is the measured isotope value of the test before any corrections, and δ^18^O_Grain boundary_ is the isotopic value of a foraminifera test in equilibrium with the ambient temperature and a δ^18^O_VSMOW_ seawater of 0‰ calculated using Eq. 8 in Bemis et al.^83^. To convert this to a temperature bias, the difference between the δ^18^O_Lattice_ and δ^18^O_Bulk_ was multiplied by 4.81 °C, which is the equivalent of a 1‰ change in δ^18^O; Eq. 8 in Bemis et al.^83^. The calculation assumes that the foraminifera tests were held in pore fluids with ambient burial temperatures that have not changed for the last 100 years, which gives enough time for the grain boundaries of each test to fully equilibrate with its pore fluids. Although the δ^18^O values of pore fluids can be altered toward higher and lower values by diagenesis/recrystallization^4^ or decrease with depth due to the presence of pore fluids from the late glacial maximum (LGM)^84–86^, we simplify our model by maintaining the pore fluid oxygen isotope composition at 0‰. Note that any calculated positive temperature bias would generally be even larger (i.e., result in even larger overestimations of the seawater paleotemperature) for benthic foraminifera tests if the modern seawater δ^18^O value was (locally) lower, as is the case for LGM pore fluids, if the geothermal gradient was steeper, and/or if the present local ambient seafloor temperature is higher than assumed in our calculations; for planktonic foraminifera tests, these trends would be in the opposite direction, i.e., resulting in even larger underestimations of the seawater paleotemperature. Furthermore, a doubling of the grain boundary volume fraction, e.g., assuming a grain boundary width of 1 nm instead of 0.5 nm, would double of the temperature bias, which makes the grain boundary volume fraction the most important parameter controlling the bias to biocarbonate seawater paleotemperature reconstructions, assuming that grain boundary diffusion is the only process acting on the oxygen isotope composition of a given biocarbonate during sediment burial.

### Supplementary information

Supplementary Information

## Data Availability

Supplementary figures are available in the Supplementary Info file. A supplementary dataset of foraminifera isotope compositions used in this manuscript is accessible through 10.6084/m9.figshare.22146800.v2.
