# Supplementary material for: Rapid grain boundary diffusion in foraminifera tests biases paleotemperature records
Source: Commun Earth Environ. 2023 Apr 27;4(1):144. doi: 10.1038/s43247-023-00798-2 (PMC11041775; doi:10.1038/s43247-023-00798-2)
Supplement: Supplementary file 1 — Supplementary Information [file 43247_2023_798_MOESM1_ESM.pdf]

Supplementary Information for:

Rapid grain boundary diffusion in foraminifera tests biases paleotemperature records

Arthur Adams,[1*] Damien Daval,[2] Lukas P. Baumgartner,[3] Sylvain Bernard,[4] Torsten Vennemann,[3] Deyanira Cisneros-Lazaro,[1] Jarosław Stolarski,[5] Alain Baronnet,[6] Olivier Grauby,[6] Jinming Guo,[1] Anders Meibom[1,7]

[1] Laboratory for Biological Geochemistry, School of Architecture, Civil and Environmental Engineering, École Polytechnique Fédérale de Lausanne (EPFL), CH-1015 Lausanne, Switzerland

[2] ISTerre, Université Grenoble Alpes, Université Savoie Mont Blanc, CNRS, IRD, IFSTTAR, 38058, Grenoble, France

[3] Institute of Earth Surface Dynamics, University of Lausanne, CH-1015 Lausanne, Switzerland

[4] Museum National d'Histoire Naturelle, Sorbonne Université, CNRS UMR 7590, IMPMC, 75005 Paris, France

[5] Institute of Paleobiology, Polish Academy of Sciences, PL-00-818 Warsaw, Poland

[6] CNRS, CINaM, Aix-Marseille Université, 13009 Marseille, France

[7] Center for Advanced Surface Analysis, Institute of Earth Science, University of Lausanne, CH-1015 Lausanne, Switzerland

* Corresponding author: arthur.adams@epfl.ch

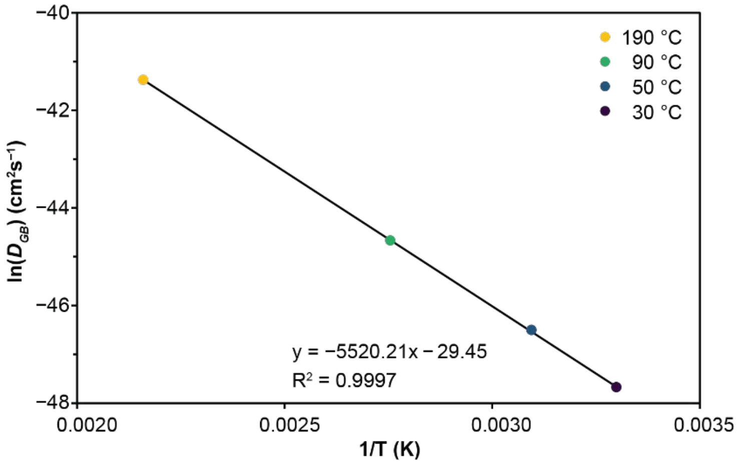

Supplementary Fig. 1 Arrhenius Plot of Oxygen Grain Boundary Diffusion Coefficients in Foraminifera Tests as a Function of Reciprocal Water Temperature.

The black line is the least squares linear regression fit (R^2 = 0.9997) to the oxygen isotope grain boundary diffusion data and its corresponding equation is shown at the bottom of the figure. The activation energy, Ea_{GB} (kJ/mol) is calculated by multiplying the slope of equation by the ideal gas constant ($J \cdot mol^{-}1 \cdot K^{-1}$) and dividing by 1000. Error bars are smaller than the symbols.

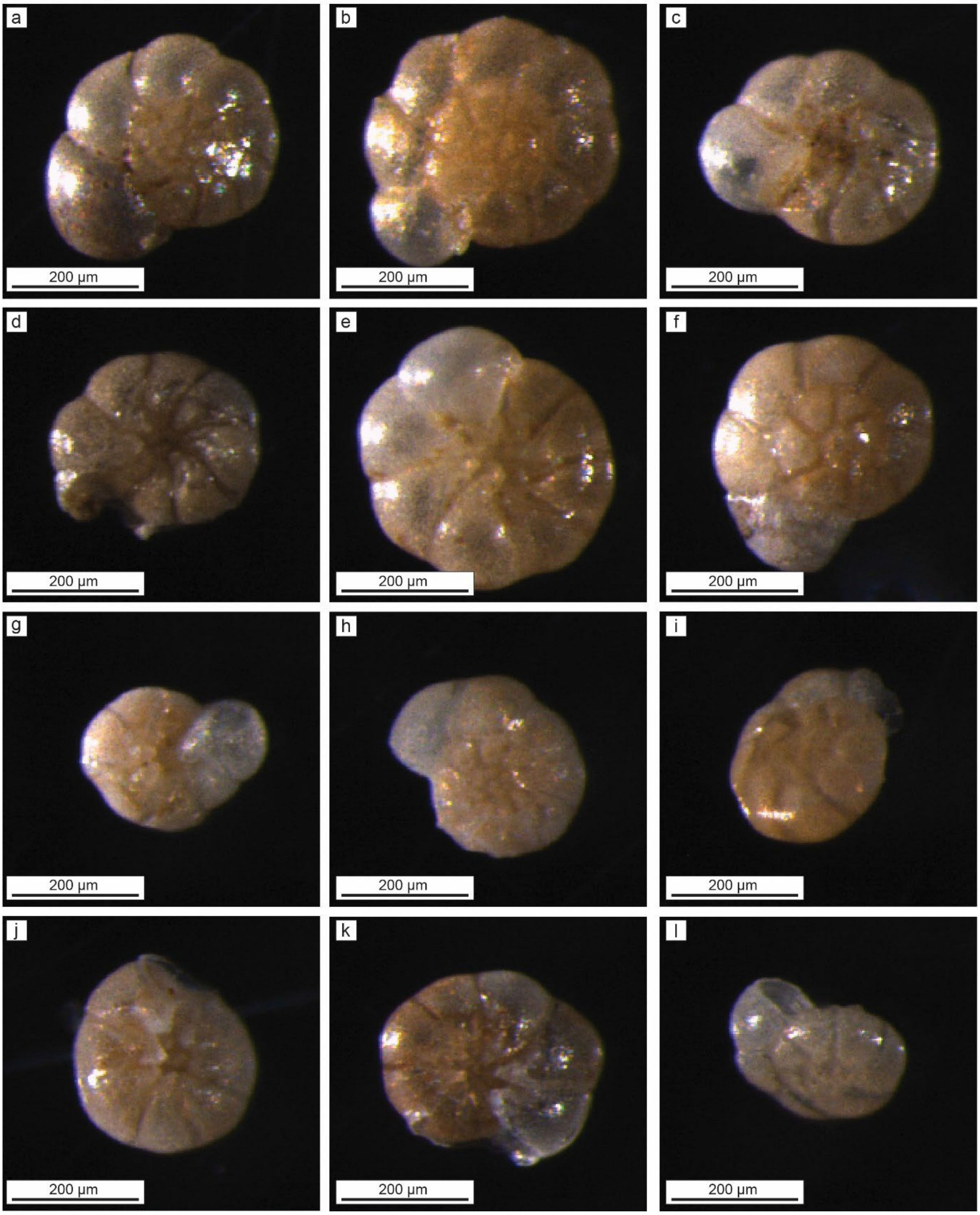

Supplementary Fig. 2 Optical images of the dorsal and ventral sides of methanol-cleaned pristine and incubated *Ammonia* sp. tests.

a–c) Methanol-cleaned tests prior to incubation. d & e) Tests incubated at 30 °C for 6 days. f & g) Tests incubated at 90 °C for 2 days. h–j) Tests incubated at 90 °C for 8 days. k & l) Foraminifera tests incubated at 190 °C for 14 days. Additional optical images of *Ammonia*

sp. tests from the same experimental conditions can be found in the supplementary figures of Cisneros-Lazaro et al.[1].

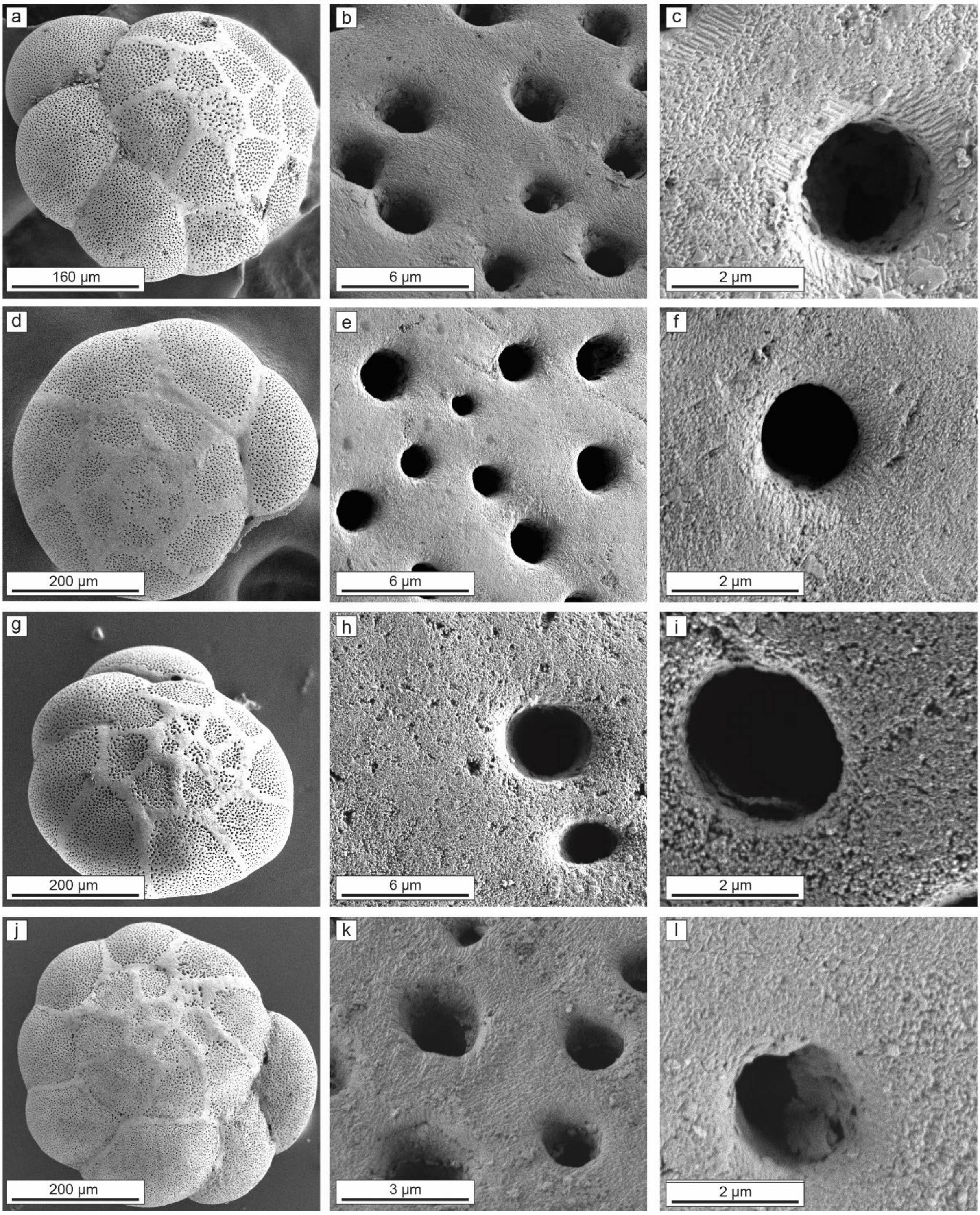

Supplementary Fig. 3 High magnification scanning electron microscopy (SEM) images of pristine and incubated *Ammonia* sp. tests.

a–c) Methanol-cleaned tests prior to incubation. d–f) Tests incubated at 30 °C for 6 days. g–i) Tests incubated at 90 °C for 8 days. j–l) Tests incubated at 190 °C for 14 days. Additional SEM images of *Ammonia* sp. tests from the same experimental conditions can be found in the supplementary figures of Cisneros-Lazaro et al.[1].

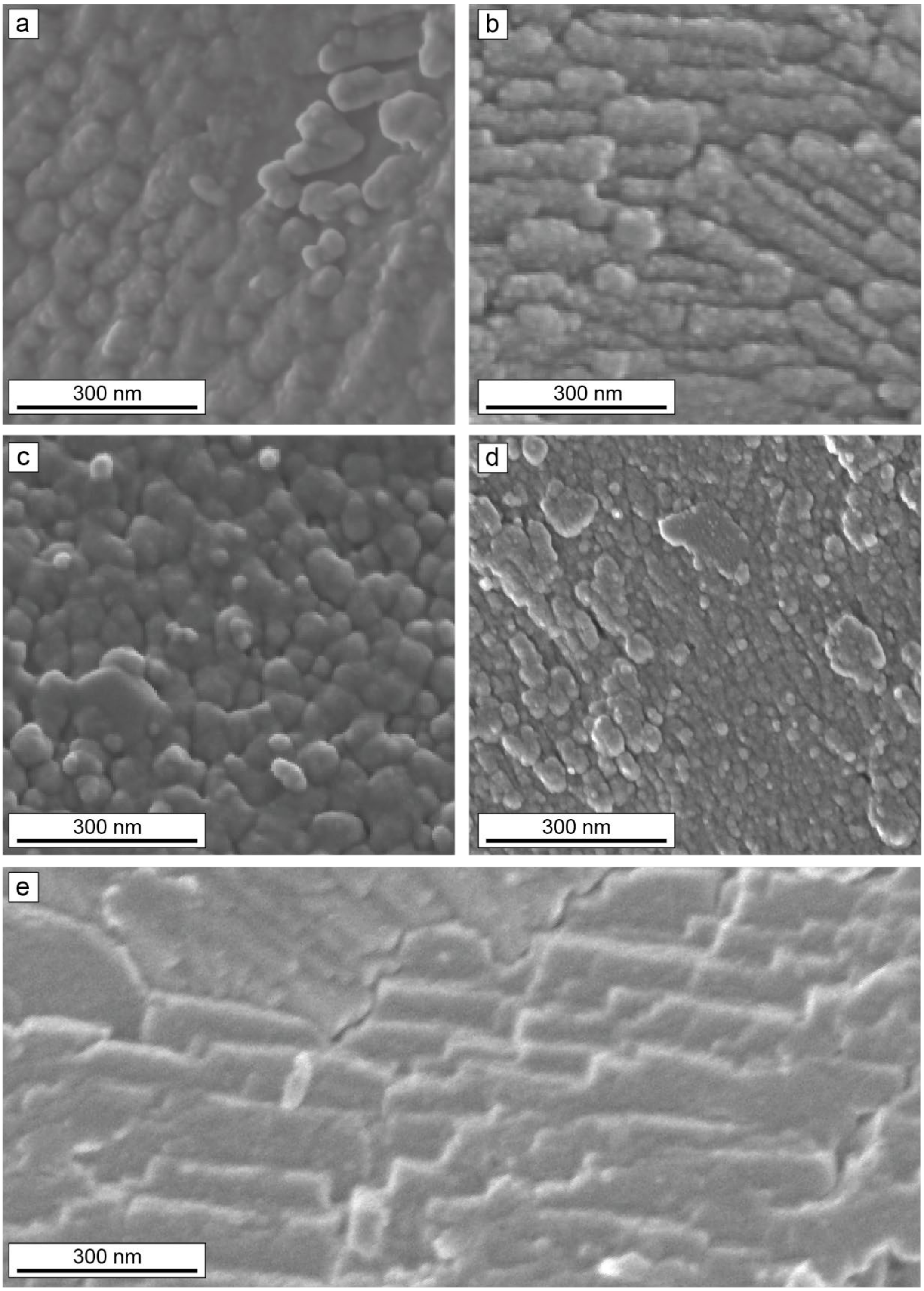

Supplementary Fig. 4 High magnification scanning electron microscopy (SEM) images of the ultrastructure of pristine and incubated *Ammonia* sp. tests.

a & b) Ultrastructure of methanol-cleaned foraminifera tests prior to incubation. c) Ultrastructure of a test after incubation at 90 °C for 3 days. d) Ultrastructure of a test after incubation at 90 °C for 9 days. e) Ultrastructure of a test after incubation at 190 °C and 14

days. Additional SEM images of *Ammonia* sp. tests from the same experimental conditions can be found in the supplementary figures of Cisneros-Lazaro et al.[1].

Table 1 Experimental Results

Experiment Type	Temp. (°C)	Time (days)	Time (√s)	Foram Weight (mg)	Fluid (μl)	Final $\delta^{18}O_{VSMOW}$ (‰)	F (%)*	O_{cal-ex}/m^2s^{-1} (moles)[1]	O_{cal-ex}/m^2 (moles)[2]	Diffusion Length (cm)
Standard	30	0.08	85	0.64	77.80	31.07 ± 0.17	0.038 ± 0.048	7.802×10^{-10}	5.618×10^{-6}	2.089×10^{-8}
Standard	30	0.25	147	0.92	55.90	31.11 ± 0.32	0.042 ± 0.055	2.922×10^{-10}	6.313×10^{-6}	2.305×10^{-8}
Standard	30	1.00	294	0.88	52.90	31.11 ± 0.69	0.042 ± 0.081	7.279×10^{-11}	6.289×10^{-6}	2.305×10^{-8}
Standard	30	3.00	509	0.72	75.60	31.33 ± 0.36	0.063 ± 0.057	3.650×10^{-11}	9.460×10^{-6}	3.494×10^{-8}
Standard	30	6.00	720	0.73	73.90	31.58 ± 0.27	0.088 ± 0.052	2.541×10^{-11}	1.317×10^{-5}	4.845×10^{-8}
Standard	30	32.00	1663	0.81	109.20	32.33 ± 0.28	0.161 ± 0.053	8.714×10^{-12}	2.409×10^{-5}	8.898×10^{-8}
Standard	50	0.08	85	0.54	91.50	31.30 ± 0.27	0.061 ± 0.052	1.262×10^{-9}	9.090×10^{-6}	3.357×10^{-8}
Standard	50	0.25	147	0.78	57.60	31.37 ± 0.63	0.068 ± 0.077	4.712×10^{-10}	1.018×10^{-5}	3.738×10^{-8}
Standard	50	0.94	285	0.94	40.30	31.62 ± 0.46	0.092 ± 0.064	1.601×10^{-10}	1.384×10^{-5}	5.099×10^{-8}
Standard	50	2.92	502	2.30	40.50	32.04 ± 0.27	0.133 ± 0.052	7.927×10^{-11}	2.000×10^{-5}	7.386×10^{-8}
Standard	50	5.90	714	2.31	40.90	32.55 ± 0.33	0.184 ± 0.056	5.398×10^{-11}	2.752×10^{-5}	1.016×10^{-7}
Standard	50	32.00	1663	0.73	54.50	33.58 ± 0.52	0.285 ± 0.068	1.547×10^{-11}	4.277×10^{-5}	1.577×10^{-7}
Standard	90	0.08	85	3.63	54.20	32.86 ± 0.38	0.217 ± 0.059	4.512×10^{-9}	3.249×10^{-5}	1.200×10^{-7}
Standard	90	0.25	147	3.87	51.10	33.62 ± 0.42	0.292 ± 0.062	2.029×10^{-9}	4.383×10^{-5}	1.619×10^{-7}
Standard	90	1.00	294	3.69	58.10	34.19 ± 0.39	0.349 ± 0.060	6.058×10^{-10}	5.234×10^{-5}	1.933×10^{-7}
Standard	90	2.17	433	2.88	54.20	34.32 ± 0.54	0.362 ± 0.071	2.895×10^{-10}	5.428×10^{-5}	2.005×10^{-7}
Standard	90	2.86	497	2.31	46.20	34.62 ± 0.41	0.392 ± 0.061	2.378×10^{-10}	5.876×10^{-5}	2.170×10^{-7}
Standard	90	6.03	722	2.40	50.20	36.09 ± 0.51	0.538 ± 0.068	1.549×10^{-10}	8.070×10^{-5}	2.980×10^{-7}
Standard	90	12.04	1020	2.84	49.60	36.37 ± 0.34	0.566 ± 0.057	8.159×10^{-11}	8.488×10^{-5}	3.135×10^{-7}
Standard	90	13.00	1060	1.32	38.30	37.09 ± 0.41	0.638 ± 0.061	8.514×10^{-11}	9.563×10^{-5}	3.531×10^{-7}
Standard	90	54.00	2160	2.49	50.50	40.81 ± 0.90	1.009 ± 0.101	3.240×10^{-11}	1.512×10^{-4}	5.582×10^{-7}
Standard	190	0.96	288	1.11	50.00	42.96 ± 0.57	1.250 ± 0.075	2.261×10^{-9}	1.872×10^{-4}	6.917×10^{-7}
Standard	190	2.92	502	1.01	77.85	47.64 ± 0.58	1.726 ± 0.075	9.980×10^{-10}	2.587×10^{-4}	9.553×10^{-7}
Standard	190	5.77	706	0.71	49.90	51.00 ± 1.04	2.068 ± 0.116	6.216×10^{-10}	3.099×10^{-4}	1.145×10^{-6}

Standard	190	8.83	873	1.22	78.70	53.59 ± 1.50	2.332 ± 0.160	1.099×10^{-8}	3.495×10^{-4}	1.291×10^{-6}
Standard	190	13.82	1093	1.02	100.50	54.76 ± 1.52	2.451 ± 0.162	7.383×10^{-9}	3.673×10^{-4}	1.356×10^{-6}
Standard	190	40.00	1859	0.89	50.00	55.03 ± 2.23	2.478 ± 0.232	1.065×10^{-10}	3.714×10^{-4}	1.372×10^{-6}
W:R Test	90	3.75	569	0.86	22.10	35.14 ± 0.48	0.444 ± 0.066	2.052×10^{-10}	6.652×10^{-5}	2.457×10^{-7}
W:R Test	90	3.76	570	0.80	62.10	35.01 ± 0.54	0.431 ± 0.071	1.990×10^{-10}	6.458×10^{-5}	2.385×10^{-7}
W:R Test	90	3.76	570	0.59	306.60	34.79 ± 0.55	0.409 ± 0.071	1.887×10^{-10}	6.130×10^{-5}	2.264×10^{-7}
Oxidized	90	0.95	286	1.47	38.40	33.62 ± 0.45	0.265 ± 0.062	2.310×10^{-9}	3.971×10^{-5}	1.466×10^{-7}
Oxidized	90	2.91	501	2.56	40.70	34.20 ± 0.37	0.323 ± 0.057	1.608×10^{-9}	4.837×10^{-5}	1.786×10^{-7}
Oxidized	90	5.90	714	0.86	40.60	34.92 ± 0.32	0.395 ± 0.053	1.380×10^{-9}	5.912×10^{-5}	2.183×10^{-7}

Experiments at 30–90 °C were conducted in artificial seawater (ASW), whereas the experiments at 190 °C were conducted in MilliQ buffered with Na_2CO_3 so that only a trace amount of calcite would dissolve before the solution reached calcite saturation. All solutions were enriched in ^{18}O to a $\delta^{18}O$ of 1000‰ VSMOW. 'Standard' experiments refer to experiments with methanol cleaned foraminifera, as described in the text. 'W:R Test' experiments were run to test the amount of isotope exchange in differing water:rock ratios. 'Oxidized' experiments consisted of experiments with oxidatively cleaned foraminifera. The fractional extent towards isotopic equilibrium (*F*) values and moles of oxygen exchanged for oxidatively cleaned foraminifera were calculated using an initial oxidatively cleaned test isotope composition of 30.96‰, whereas all other aliquots used the value of methanol cleaned foraminifera of 30.68‰. *Fractional extent towards isotopic equilibrium. [1]Moles of oxygen exchanged between the fluid and the calcite normalized to the sample run time and aliquot mass. [2]Moles of oxygen exchanged between the fluid and the calcite normalized to the sample run time. Surface area normalized values and diffusion lengths were calculated using a specific surface area[2] of 2 m^2/g.

References

1. Cisneros-Lazaro, D. *et al.* Fast and pervasive diagenetic isotope exchange in foraminifera tests is species-dependent. *Nat. Commun.* **13**, 113 (2022).
2. Honjo, S. & Erez, J. Dissolution rates of calcium carbonate in the deep ocean; an in-situ experiment in the North Atlantic Ocean. *Earth Planet. Sci. Lett.* **40**, 287–300 (1978).
